# Nanoparticle Detection of Urinary Markers for Point-of-Care Diagnosis of Kidney Injury

**DOI:** 10.1371/journal.pone.0133417

**Published:** 2015-07-17

**Authors:** Hyun Jung Chung, Kathryn L. Pellegrini, Jaehoon Chung, Kamani Wanigasuriya, Innocent Jayawardene, Kyungheon Lee, Hakho Lee, Vishal S. Vaidya, Ralph Weissleder

**Affiliations:** 1 Center for Systems Biology, Massachusetts General Hospital, 185 Cambridge St, CPZN 5206, Boston, Massachusetts, United States of America; 2 Renal Division, Department of Medicine, Brigham and Women’s Hospital, Harvard Medical School, Boston, Massachusetts, United States of America; 3 Department of Medicine, Faculty of Medical Sciences, University of Sri Jayewardenepura, Nugegoda, Sri Lanka; 4 Channing Laboratory, Department of Medicine, Brigham and Women's Hospital, Harvard Medical School, and Harvard School of Public Health, Boston, Massachusetts, United States of America; 5 Department of Environmental Health, Harvard School of Public Health, Boston, Massachusetts, United States of America; 6 Department of Systems Biology, Harvard Medical School, 200 Longwood Ave, Boston, Massachusetts, United States of America; University of Utah School of Medicine, UNITED STATES

## Abstract

The high incidence of acute and chronic kidney injury due to various environmental factors such as heavy metals or chemicals has been a major problem in developing countries. However, the diagnosis of kidney injury in these areas can be more challenging due to the lack of highly sensitive and specific techniques that can be applied in point-of-care settings. To address this, we have developed a technique called ‘micro-urine nanoparticle detection (μUNPD)’, that allows the detection of trace amounts of molecular markers in urine. Specifically, this technique utilizes an automated on-chip assay followed by detection with a hand-held device for the read-out. Using the μUNPD technology, the kidney injury markers KIM-1 and Cystatin C were detected down to concentrations of 0.1 ng/ml and 20 ng/ml respectively, which meets the cut-off range required to identify patients with acute or chronic kidney injury. Thus, we show that the μUNPD technology enables point of care and non-invasive detection of kidney injury, and has potential for applications in diagnosing kidney injury with high sensitivity in resource-limited settings.

## Introduction

The occurrence of acute kidney injury (AKI) and chronic kidney disease (CKD) has been a major problem in poor, rural, agricultural communities in parts of Central America, Sri Lanka, and India [1]. The exact etiology of CKD in these communities still remains a mystery. However, it has been suggested that exposure to various environmental factors such as heavy metals, pesticides, other chemicals or plant products are associated with high prevalence of CKD, eventually requiring dialysis or kidney transplantation [2–4]. The traditional biomarkers of CKD and AKI are blood urea nitrogen (BUN), serum creatinine (SCr), and urinary albumin, markers which have been in place in clinical practice for decades but have limitations with respect to sensitivity, specificity, and timeliness of diagnosis [5,6]. Serum biomarker measurements also require drawing blood from patients, with repeated sampling when the diagnosis results are positive. Thus, the procedure can be challenging, costly and impractical in rural areas. Therefore, low-cost, non-invasive techniques using urine which allow point-of-care analyses are highly desirable.

The detection of urinary rather than serum markers has provided a major advantage in monitoring AKI due to the non-invasiveness and simplicity of the sampling procedure [7–9]. Soluble proteins or other biomolecules, such as kidney injury molecule-1 (KIM-1), Cystatin C, and neutrophil gelatinase-associated lipocalin (NGAL) have been reported to be present at high levels in the urine of patients with acute or chronic kidney injury [10–13]. However, most of these studies have utilized complex instruments (e.g. Luminex), which have high sensitivity but are impractical for rural testing. A test-strip method based on colorimetric detection was developed, and would have been more useful for diagnoses in point-of-care or home settings, but lacked a quantitative read-out system and the required sensitivity [14].

Recently, magnetic nanoparticle-based diagnostics have shown broad utility for the detection of different human diseases such as cancer [15,16] and infectious diseases [17,18]. Diagnostic targets, in the form of cells or extracted molecular components, are labeled with magnetic nanoparticles which are then detected by a miniaturized nuclear magnetic resonance device (μNMR). Not only the device is portable allowing diagnosis in resource-limited settings, but also the detection based on magnetic resonance is highly sensitive due to the low background signals. In addition, the utilization of nanoparticles increases the molecular density of ligands, enabling rapid and efficient labeling of disease targets [19,20]. The method can be integrated into an on-chip, automated system, and exhibited extremely high sensitivity allowing the detection of single cell targets from complex biological fluids such as whole blood [21].

Here, we describe the development of a semi-automated, nanoparticle labeling procedure designed to detect soluble protein markers KIM-1 and Cystatin C in urine, which we have termed micro-urine nanoparticle detection (μUNPD). KIM-1 is a type I cell membrane glycoprotein which upon acute kidney injury, is highly expressed in the apical membrane of proximal tubules and releases its ectodomain into the urine [11]. Cystatin C is an inhibitor of cysteine proteases which is expressed in all human nucleated cells, and has been shown to be upregulated in various pathological conditions including states of chronic and acute kidney injury [13]. We assessed the feasibility of the technology by measuring limit of detection for both markers, as well as testing clinical samples from 42 different individuals that were either patients with kidney disease or healthy volunteers. We found that our miniaturized magnetic detection method, μUNPD, provided quantitative read-out signals with high sensitivity.

## Methods

### Microfluidic device fabrication and assembly

The microfluidic devices were fabricated by stacking three layers of polydimethylsiloxane (PDMS; Dow Corning) on a glass slide. The cast molds for the bottom and top layers were prepared by patterning a 50 μm thick single layer of epoxy-based SU8-3050 photoresist (Microchem). A filter PDMS block was separately prepared by sandwiching a membrane filter (400 nm pore diameter: Nuclepore, Whatman) with two PDMS slabs. Prior to assembly of the block, the membrane filter was cut into a circular shape (5 mm in a diameter), 1mm thick PDMS slabs were diced into 7×7 mm^2^ and a hole (2.5 mm in a diameter) was punched in the center for vertical connection between bottom and upper channels through the filter. Then, the three parts were aligned and bonded using uncured PDMS as a glue. The channel patterns were replicated into the bottom layer (thickness, 1 mm) by pouring and curing PDMS, while additional fluidic channels were contained within the separate top PDMS layer (thickness 1 mm). In the bottom PDMS layer a hole (2 mm in a diameter) were generated to connect the bottom fluidic channel to filter and upper channel. Then, the PDMS filter block was aligned and glued on top of the bottom layer. Uncured PDMS polymer was then poured over the assembly, forming the intermediate layer (final thickness, 2 mm). After the polymer had cured, the top PDMS layer was then irreversibly bonded to the intermediate layer via oxygen plasma treatment. Inlet and outlet reservoirs were punched out, and the assembled device was finally bonded irreversibly to a glass slide.

### Preparation of assay reagents

For preparation of the capture beads, 1 mg of carboxylated polystyrene beads (diameter, 3 μm; Polysciences) were reacted with 1.92 mg of 1-ethyl-3-[3-dimethylaminopropyl]carbodiimide hydrochloride (EDC, Sigma-Aldrich) and 1.15 mg of *N*-hydroxysulfosuccinimide (sulfo-NHS, Thermo Scientific) in phosphate buffered saline (PBS, pH 7.4) for 4 hr at room temperature. After washing the unreacted chemicals using centrifugal filters (0.45 μm, Millipore), the beads were then added with 100 μg of goat polyclonal antibody to human KIM-1 (R&D Systems) or goat polyclonal antibody to human Cystatin C (R&D Systems), and reacted for 12 hr in PBS with 10 mM sodium bicarbonate at room temperature. The beads were finally washed with centrifugal filters to remove the unreacted antibody. For preparing the detection antibody conjugates (Ab-TCO), 100 μg of polyclonal antibody to KIM-1 or Cystatin C in PBS including 10 mM sodium bicarbonate were added with 94 μg of trans-cyclooctene N-hydroxy-succinimidyl ester (TCO-NHS) in dimethylformamide (molar ratio 1:500) and reacted for 4 hr in room temperature. The antibodies were purified using Zeba columns (MWCO 7,000, Thermo Scientific). The magnetic nanoparticle conjugates (MNP-Tz) were prepared by adding 0.5 mg of amine-modified iron oxide nanoparticles (diameter, 5 nm or 30 nm; Ocean Nanotech) in PBS including 10 mM sodium bicarbonate with 472 μg of 2,5-dioxopyrrolidin-1-yl 5-(4-(1,2,4,5-tetrazin-3-yl)benzylamino)-5-oxopentanoate (tetrazine-NHS) in dimethylsulfoxide and reacted at room temperature for 4 hr. The nanoparticles were purified using centrifugal filters (Amicon Ultra, MWCO 100,000; Millipore).

### Clinical samples

Ethical approval for the clinical studies were obtained from the Harvard School of Public Health Institutional Review Board (IRB), Brigham and Women’s Hospital (BWH) Office of Human Research Administration, and the Faculty of Medical Sciences, University of Sri Jayawardenepura, Sri Lanka with permission from The Ministry of Health, Colombo, Sri Lanka. All subjects were approached after obtaining verbal consent from the physician of record to describe the study. Subjects were informed that participation is entirely voluntary and will no way affect their medical care. Written informed consent was obtained from all participants. The respective institutional review board approved the written consent prior to the start of the study.

#### Sri Lankan cohort—Chronic kidney disease (CKD)

A subset of samples collected for urinay biomarker and toxic metal evaluation in CKD patients and controls were used for this study. Urine samples (n = 18) were collected from individuals with CKD patients attending a renal clinic at Medawachchiya hospital in the North Central Province of Sri Lanka. Patients with CKD had serum creatinine levels greater than 2 mg/dL, with an estimated glomerular filtration rate (eGFR) lower than 60 ml/min/1.73m^2^. A group of 18 healthy individuals from the Western Province in Sri Lanka who are not engaged in farming and living in the endemic area likely to be exposed to same environmental risk factors were invited to participate as controls as healthy individuals.

#### BWH cohort—Acute kidney injury (AKI)

Urine samples were collected from ICU patients (n = 3) at Brigham and Women’s Hospital (BWH) with approval of the IRB of BWH. AKI was defined as an increase of serum creatinine of at least 100% over baseline values. Urine samples were also collected from 3 healthy volunteers as controls.

### Magnetic nanoparticle assay (μUNPD)

**Fig 1:**
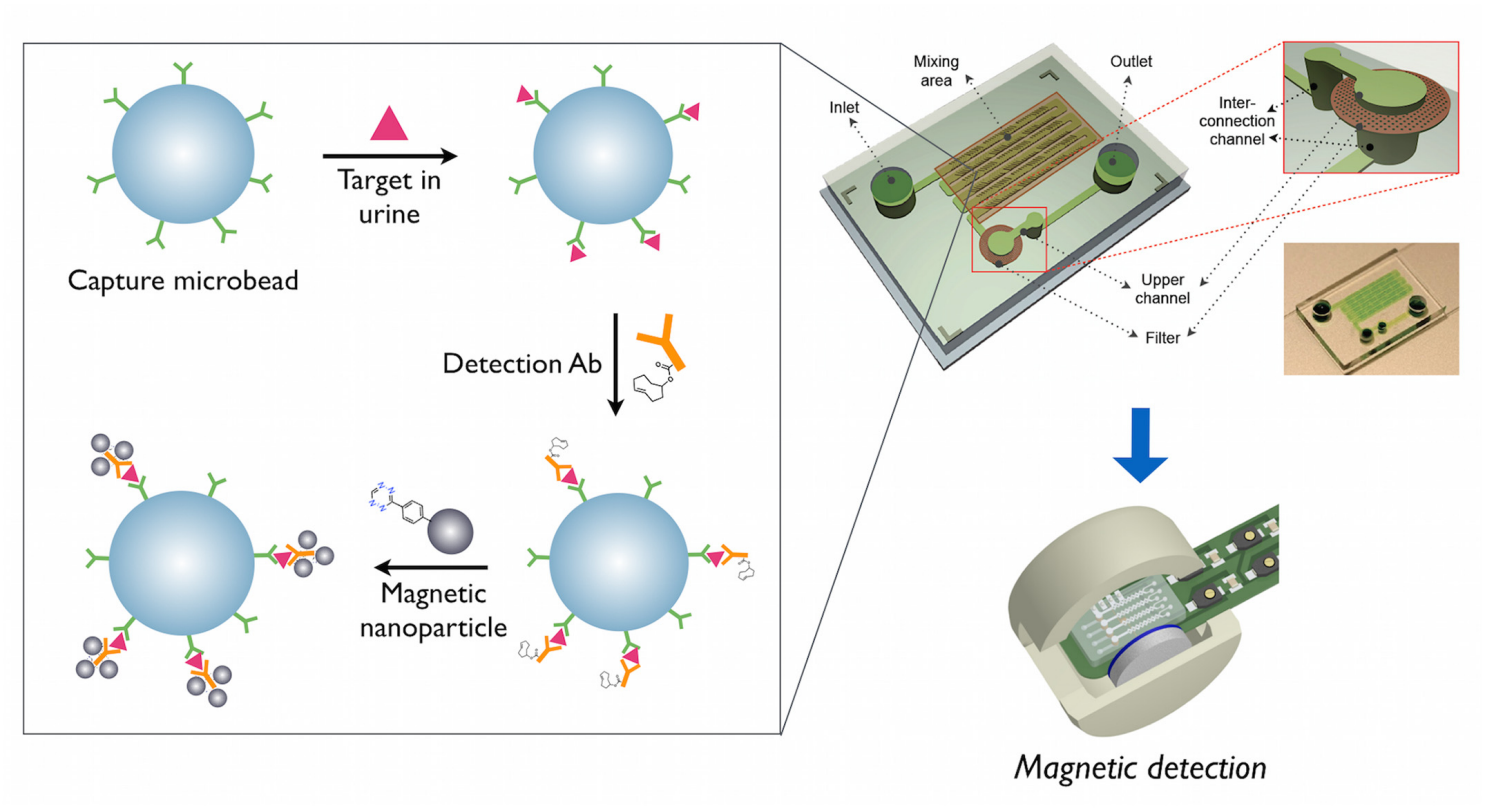
Schematic illustration of the μUNPD assay for urinary marker detection. Capture antibody conjugated-microbeads, detection antibodies (TCO conjugated), and magnetic nanoparticles (Tz conjugated) are sequentially added to the urine specimen in a microchip, for subsequent read-out measurements using the μNMR device.

For performing the magnetic immuno-sandwich assay in high-throughput, the capture beads for either KIM-1 or Cystatin C were added with the target solution in micro-well filter plates (Multiscreen, Millipore) and incubated at room temperature for 45 min. Unbound target was removed by washing with PBS including 0.05% Tween (PBS-T) and suction using a vacuum manifold (Millipore). The capture beads were then added with the detection antibody conjugates (Ab-TCO) for KIM-1 or Cystatin C in PBS including 1 mg/ml bovine serum albumin and 2% fetal bovine serum (PBS++), incubated at room temperature for 45 min, and washed with PBS-T. Finally, the magnetic nanoparticles (MNP-Tz) were added at 50 μg/ml for the 30 nm (KIM-1) or 100 μg/ml for 5 nm (Cystatin C) particles in PBS++ and incubated for 20 min at room temperature, followed by washing with PBS-T. For the automated, on-chip procedure, the capture beads were added in the inlet of the micro-chip (Fig 1). The target solution was also added into the inlet, and the beads were allowed to mix with the target by flowing through the micro-channels by applying negative pressure. Washing solution (PBS-T) was added to the ‘outlet’ and ‘inlet’, alternatively. The detection antibody conjugates (Ab-TCO) and magnetic nanoparticles (MNP-Tz) were also sequentially applied to the inlet of the micro-chip, with washing steps performed in between as described above. As the target solution, either the purified recombinant protein of KIM-1 or Cystatin C in buffer solution, or the clinical samples (undiluted for KIM-1, and 5x diluted in PBS++ for Cystatin C) were added to the capture beads.

After the assay, samples were reconstituted in 10 ul PBS and applied into polyimide tubings for T2 relaxation measurements using a miniaturized nuclear magnetic resonance device [22]. Transverse relaxation times were measured using Carr–Purcell–Meiboom–Gill pulse sequences with the following parameters: echo time, 3 ms; repetition time, 4 s; number of 180° pulses per scan, 900; number of scans, 7. All measurements were done in triplicate, and data are expressed as mean ± standard deviation (SD). Limit of detection (LOD) values were determined by calculating 3×(SD of background signal).

### Luminex assay

Luminex xMAP technology was used to measure urinary Cystatin C with the Human Kidney Toxicity Multiplex Assay from Millipore according to manufacturer instructions, and urinary kidney injury molecule-1 (KIM-1) was measured using a previously established Luminex-based assay [7,12]. Urinary creatinine concentration was measured using a kit from Cayman Chemicals according to manufacturer instructions, and used to normalize protein biomarker measurements to account for urinary volume differences.

## Results

The μUNPD technology is currently a semi-automated, microchip-based nanoparticle detection method to measure trace amounts of biomarker proteins in urine. Fig 1 shows the overall scheme of the μUNPD assay. First, microbeads coated with capture antibodies are incubated with the urine sample, which contains the target protein. The unbound molecular components are washed away and the detection antibody is added. After another washing step, the magnetic nanoparticles (MNPs) are added for labeling, followed by the final washing step, and the labeled beads are applied to the μNMR for *T*_2_ relaxation measurements. A bioorthogonal reaction using trans-cyclooctene and tetrazine was introduced for chemically linking the detection antibodies with the MNPs. All assay components were added to the inlet of the microfluidic chip. On-chip channels allowed effective mixing, while a filter membrane acted as a barrier to trap the beads while the solutions would flow through. The entire procedure was simple and straightforward, taking only two hours to complete.

### Validation of assay

**Fig 2:**
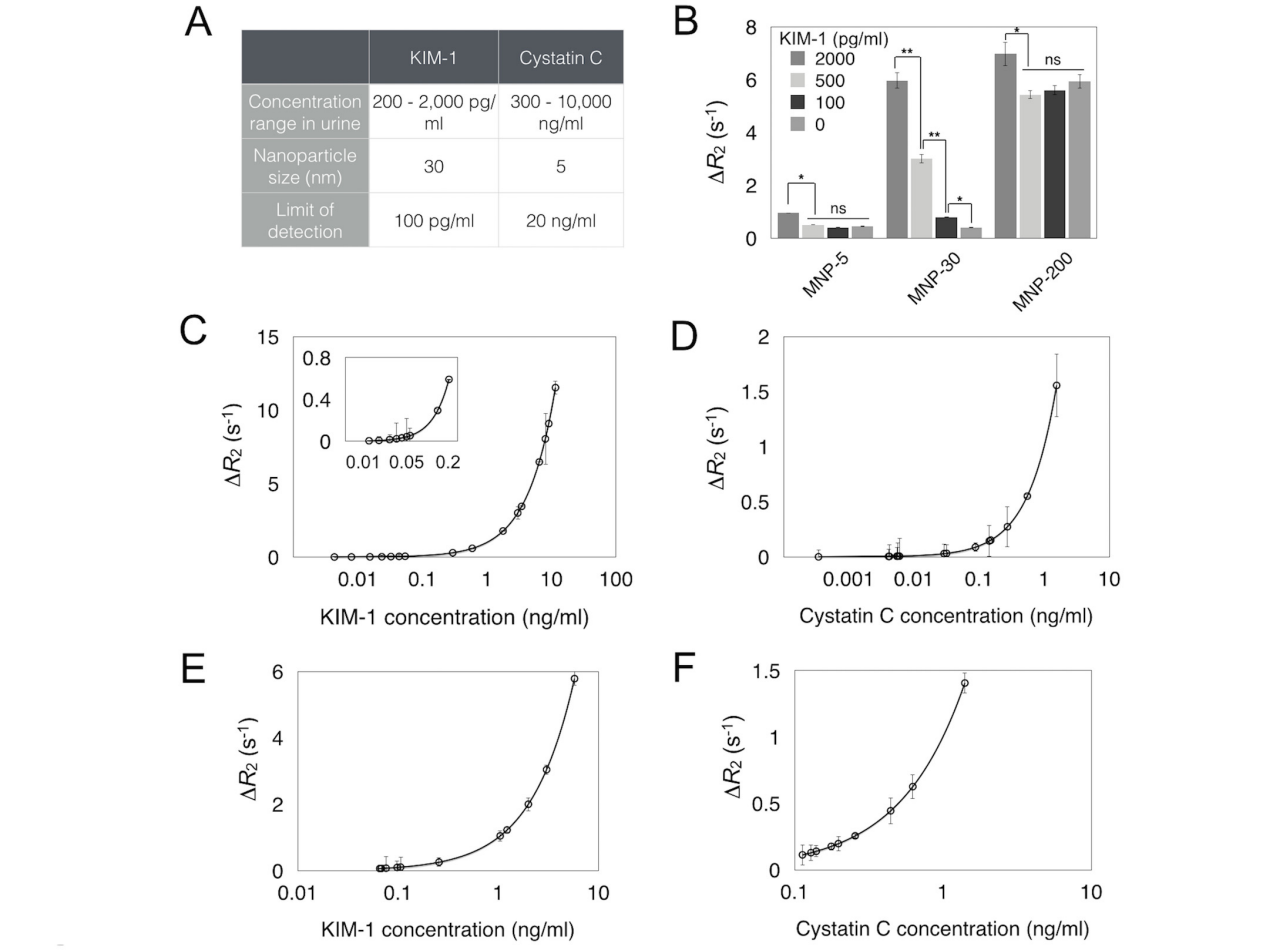
Assay validation. A, Assay set-up for urinary marker detection. B, μNMR signals using MNPs with different sizes. * = *p* < 0.02, ** = *p* < 0.001, ns = not significant. Detection sensitivity measurements using serial dilutions of recombinant KIM-1(C) and Cystatin C (D) in buffer solution. Inset in C shows data points in low ranges of KIM-1. ΔR2 = R2 (sandwich)—R2 (bead only). E,F, Detection of KIM-1 (E) and Cystatin C (F) in 100% and 20% urine, respectively. The urine used was from a healthy patient (no. 20) with ranges of KIM-1 and Cystatin C that were not detectable. Note that all the clinical samples (in Fig 3) were analyzed based on the same dilution factors.

The feasibility of the μUNPD assay was first validated with recombinant proteins of the urinary markers spiked into urine or buffer solutions. Fig 2A shows the detection ranges of each marker, with optimum sizes of the magnetic nanoparticles used for labeling. For KIM-1, a robust concentration dependent response was observed with 30 nm magnetic nanoparticles which had higher relaxivity values than 5 nm nanoparticles (Fig 2B). Larger nanoparticles with a size of up to 200 nm showed much higher background signals, resulting in lower detection sensitivity than the 30 nm nanoparticles. Detection sensitivity of the μUNPD method was assessed by spiking in different amounts of recombinant protein into the buffer solution prior to performing the assay. In contradistinction, Cystatin C can be present at much higher concentrations and therefore smaller nanoparticles can be used. Fig 2C and 2D shows that the lower detection limits of KIM-1 and Cystatin C were 100 pg/ml and 20 ng/ml, respectively. The proteins were also spiked into urine from a healthy individual, which showed similar signals compared to detection in buffer solution (Fig 2E and 2F). These detection limits are sufficient to detect kidney injury.

### Clinical testing

**Fig 3:**
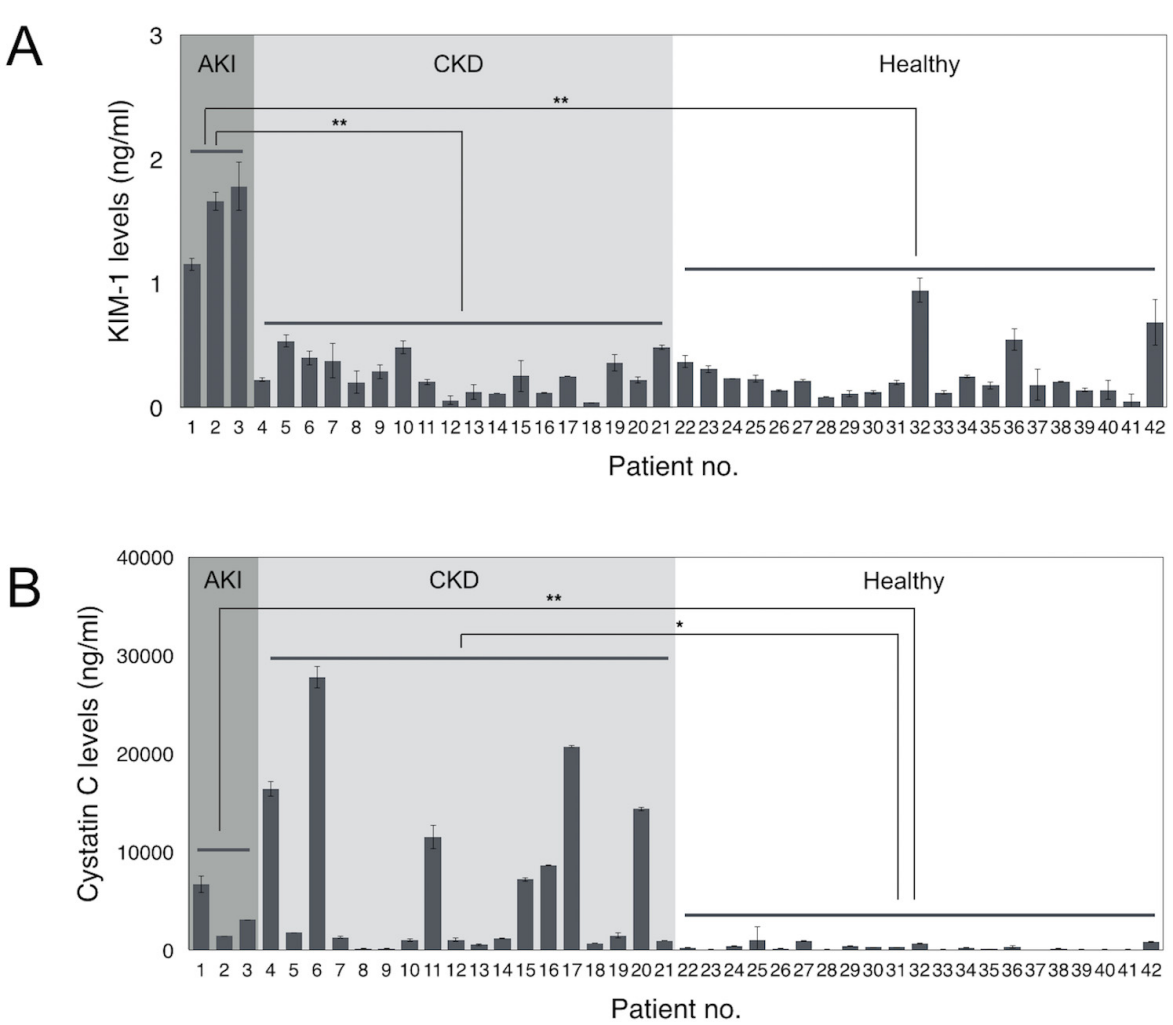
Measurement of KIM-1 and Cystatin C in clinical samples using the μUNPD technology. Urine samples were obtained from 21 patients with kidney injury (AKI, acute kidney injury, n = 3; CKD, chronic kidney disease, n = 18) as well as 21 healthy individuals as controls. (A) KIM-1 levels were measured and expressed based on the calibration from S1A Fig, without any dilution (100% urine). (B) Cystatin C levels were measured and expressed based on calibration from S1B Fig, after 5X dilution in PBS (20% urine). * = *p* < 0.002, ** = *p* < 0.0001.

**Fig 4:**
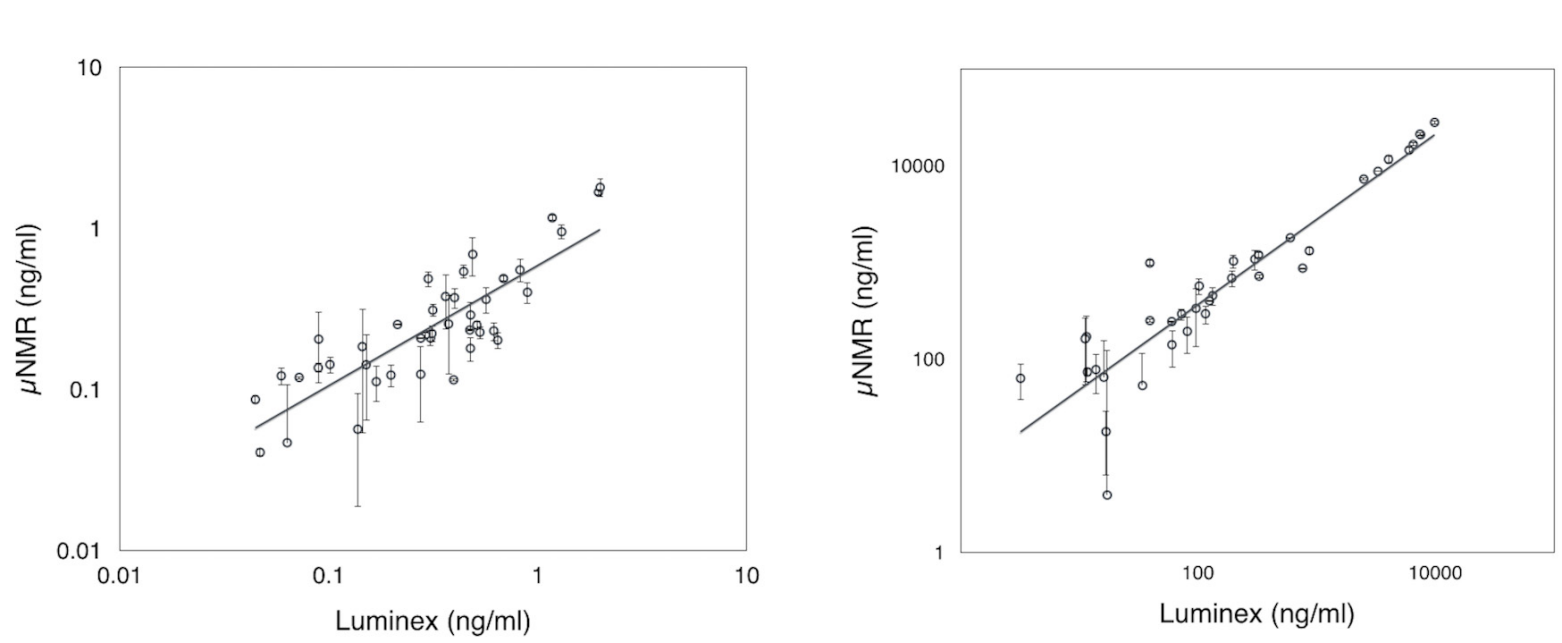
Correlation between μUNPD & luminex method. Detection of KIM-1 (A) and Cystatin C (B). Levels of biomarker proteins for μUNPD and luminex were obtained from standard calibration curves from each method, individually.

We next evaluated the clinical utility of the μUNPD assay using urine specimens from a mixed cohort of 42 patients. The majority of this cohort was comprised of a group of Sri Lankan farmers that had been diagnosed with chronic kidney disease (CKD; n = 18) or their age-matched controls working in the same area that had no such diagnosis (n = 18). In addition, we included samples from a group of ICU patients at BWH that had been diagnosed with acute kidney injury (AKI; n = 3) so that we could observe the higher limits of KIM-1 detection, as well as control samples from healthy volunteers (n = 3) (Table 1). Fig 3A and 3B show the detected levels of KIM-1 and Cystatin C in each sample, respectively. Protein concentrations were calculated based on calibrations from measuring spiked recombinant proteins (S1 Fig). All 21 controls showed low levels of both KIM-1 and Cystatin C. All three patients in the AKI cohort showed extremely high levels of KIM-1, while also showing a significant levels of Cystatin C. KIM-1 was not found to be significantly elevated in any of the 18 patients from the CKD cohort, whereas Cystatin C was elevated in 16 of these patients. Combined tests of KIM-1 and Cystatin C had a specificity of 89% for all kidney injuries. These results were in agreement with the measurements of KIM-1 and Cystatin C using a Luminex-based assay and confirm that KIM-1 is a useful biomarker for AKI, while Cystatin C is associated with both AKI and CKD. A direct comparison of measurements taken with the μUNPD method and luminex-based assay showed good correlation for both biomarkers (Fig 4A and 4B; R^2^ = 0.8641 for KIM-1, R^2^ = 0.9936 for Cystatin C).

**Table 1:** Human subjects.

Cohort		Age, years	SCr, mg/dL	eGFR, ml/min/1.73m^2^
**Sri Lankan cohort**	CKD, n = 18	56.8 (7.1)^a^	4.1 (2.2)	20.6 (9.6)
	Healthy, n = 18	57.3 (8.6)	1.0 (0.1)	82.4 (15.0)
**BWH cohort**	AKI, n = 3	48.0 (33.0)	2.6 (1.7)	N/A
	Healthy, n = 3	24.0 (3.5)	N/A	N/A

^a^Data presented as mean (SD).

## Discussion

We show proof-of-principle that the magnetic μUNPD technology can be used to measure kidney injury biomarkers KIM-1 and Cystatin C in urine samples using a handheld device. The technology enables the detection of extremely low amounts of biomarkers present in the urine. The intensity of read-out signals, which are *T*_2_ relaxation values, can be controlled by changing the size of labeling material which vary in *r*_2_ relaxivity values. Larger particles have a higher core-to-shell volume ratio resulting in higher relaxivity. The larger volume and perhaps surface impurities leads to higher nonspecific binding. Using the biomarkers KIM-1 and Cystatin C, the μUNPD method allowed the diagnoses and discrimination of both AKI and CKD in patients via a non-invasive procedure. The results from the clinical studies correlate well with the previous reports that KIM-1 is indicative of AKI, while Cystatin C can be a generic marker for both acute and chronic injury [3].

A dipstick method for KIM-1 detection has previously been reported but showed a detection limit of 0.8 ng/ml [14]. The μUNPD method, on the other hand, was able to quantitatively detect KIM-1 with a lower detection limit of 0.1 ng/ml. High sensitivity is a key feature of magnetic detection, since *T*_2_ relaxation measurements based on magnetic nanoparticles show highly intense signals with very low background and nonspecific signals, compared to existing methods based on optical detection such as fluorescence measurements. Sensitivity is critical, since KIM-1 is commonly present in urine at concentrations down to 0.1 ng/ml. We found the detection sensitivity of our handheld point-of-care device to be comparable to the Luminex-based assay [7], and thus clearly has financial and practical advantages for regions with limited resources (equipment costs ~ $120,000 for Luminex technology vs ~ $1,000 for μUNPD technology).

Magnetic detection of disease targets based on nanoparticle labeling has been reported in several studies previously [15,17,18]. However, this study is the first to 1) detect soluble markers, 2) directly from a patient specimen without any purification or processing steps, and 3) through a non-invasive sampling procedure. The further identification of soluble urinary biomarkers in other diseases such as cancer and infections would greatly broaden the applicability of the μUNPD technology.

The utilization of bioorthogonal linkers also contributes to the high read-out signals and rapidness of the procedure. The reaction between trans-cyclooctene and tetrazine is an ultra-fast reaction with a second-order rate constant of 6,000 ± 200 M^-1^s^-1^ [23]. The small size of the linkers also endows high molecular density of the linker on the magnetic material and ligand, allowing their efficient and rapid binding to the target.

Finally, the automation of the assay in a micro-chip format reduces the need for training to perform the assay, increasing the applicability of this approach as a point-of-care diagnostic. We have shown here that KIM-1 and Cystatin C can be accurately and sensitively detected in urine samples with our technique, and we envision that assays for other urinary markers, such as NGAL, Clusterin and albumin, could also be easily adapted to our method. An extended panel of markers would create a more informed platform, to further contribute in decision-making and personalized treatment options. Although the measurements were obtained separately for each marker in the current study, strategies to multiplex the assay are being explored. Multiplexing would greatly improve the throughput of the assay and perhaps further reduce costs. In addition, urine-based diagnostics can be complicated by the fact that variable amounts of urine are produced among individuals. Advanced methods to normalize biomarker levels should be further developed to minimize these complexities.

## Conclusions

The current study presents a semi-automated, point-of-care method that can detect biomarkers in urine samples. The method is based on capture and nanoparticle-labeling of target proteins followed by detection using a hand-held magnetic detection device. The technology is highly sensitive, comparable to the standard Luminex-based assay, with low background signals, and simple which would allow its easy application for clinical diagnosis. The utilization of urinary biomarkers KIM-1 and Cystatin C also provides advantages in maximizing specificity and endowing simplicity in the procedure.

The occurrence of acute or chronic kidney injury due to various environmental factors has been a major problem in poor, rural communities in developing countries. Most of these locations are limited in resources and poorly accessible to hospital facilities, requiring a simple, point-of-care technology for diagnosing kidney injury. The μUNPD technology, which is much more sensitive than the previously developed dipstick method, will greatly benefit these communities in identifying patients’ conditions and allowing subsequent treatment procedures.

## Supporting Information

S1 Fig Conversion of NMR signals. Calibration plot to convert NMR signals into KIM-1 (A) and Cystatin C (B) levels in the clinical samples.
(DOCX)
