# Supplementary material for: Nanoparticle Detection of Urinary Markers for Point-of-Care Diagnosis of Kidney Injury
Source: PLoS One. 2015 Jul 17;10(7):e0133417. doi: 10.1371/journal.pone.0133417 (PMC4506142; doi:10.1371/journal.pone.0133417)
Supplement: S1 Fig — Calibration plot to convert NMR signals into KIM-1 (A) and Cystatin C (B) levels in the clinical samples. (DOCX) [file pone.0133417.s001.docx]

**Supporting Information**

**Nanoparticle detection of urinary markers for point-of-care diagnosis of kidney injury**

Hyun Jung Chung^1,†,#a^, Kathryn L. Pellegrini^2,†^, Jaehoon Chung^1,†^, Kamani Wanigasuriya^3^, Innocent Jayawardene^4^, Kyungheon Lee^1^, Hakho Lee^1,^, Vishal S. Vaidya^2,5^, Ralph Weissleder^1,6,^*

^1^ Center for Systems Biology, Massachusetts General Hospital, 185 Cambridge St, CPZN 5206, Boston, MA 02114, USA

^2^ Renal Division, Department of Medicine, Brigham and Women’s Hospital, Harvard Medical School, Boston, MA 02115, USA

^3^ Department of Medicine, Faculty of Medical Sciences, University of Sri Jayewardenepura, Nugegoda, Sri Lanka

^4^ Channing Laboratory, Department of Medicine, Brigham and Women's Hospital, Harvard Medical School, and Harvard School of Public Health, Boston, MA 02115, USA

^5^ Department of Environmental Health, Harvard School of Public Health, Boston, MA 02115, USA

^6^ Department of Systems Biology, Harvard Medical School, 200 Longwood Ave, Boston, MA 02115, USA

^#a^ Current Address: Graduate School of Nanoscience and Technology, Korea Advanced Institute of Science and Technology, Daejeon 305-701, Republic of Korea

^†^ Authors contributed equally

*Corresponding author

R. Weissleder, MD, PhD

Center for Systems Biology

Massachusetts General Hospital

185 Cambridge St, CPZN 5206

Boston, MA, 02114

617-726-8226

[rweissleder@mgh.harvard.edu](mailto:weissleder@helix.mgh.harvard.edu)


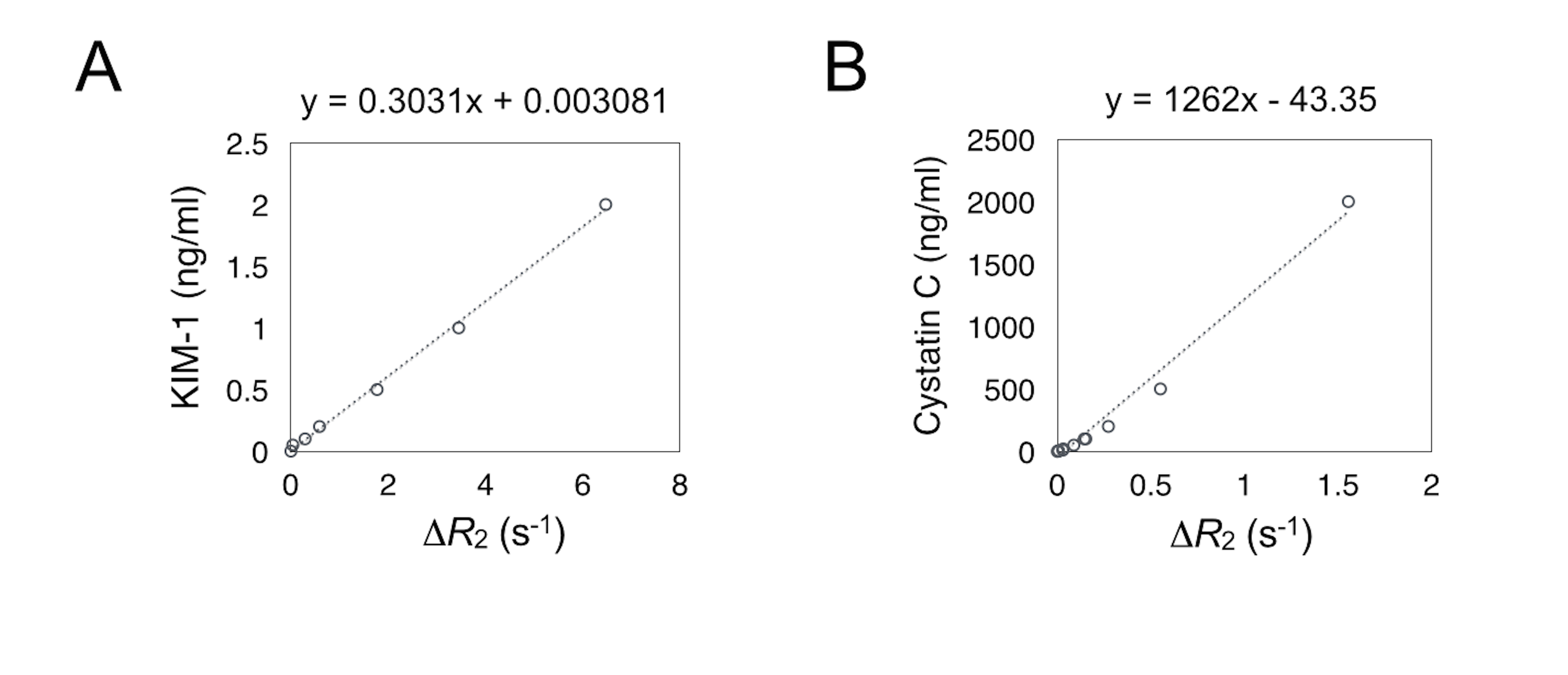


Supplemental Figure 1. Calibration plot to convert NMR signals into KIM-1 (A) and Cystatin C (B) levels in the clinical samples.
